# Balancing the scales: predictors of performance and the long-term impact of a robotic surgery curriculum

**DOI:** 10.1007/s00464-026-13035-6

**Published:** 2026-07-07

**Authors:** Colin M. Johnson, Sarah B. Hays, Jason L. Schwarz, Kristine Kuchta, Aram Rojas, Syed A. Mehdi, Sangrag Ganguli, Alessia Vallorani, Miral S. Grandhi, Melissa E. Hogg

**Affiliations:** 1 Department of Surgery, Rutgers New Jersey Medical School, Newark, NJ USA; 2 Department of Surgery, University of Chicago, Chicago, IL USA; 3 Department of Surgery, Endeavor Health – Evanston Hospital, 2650 Ridge Ave, Chicago, IL 60201 USA; 4 Division of Pancreatic and Transplant Surgery, Pancreas Translational and Clinical Research Center, IRCCS San Raffaele Scientific Institute, Milan, Italy

**Keywords:** Resident education, Robotic surgery training, Simulation-based training, Virtual reality simulation, OSATS, Robotic surgery curriculum

## Abstract

**Background:**

The expansion of robotic surgery has created interest in resident robotic curricula to develop competent robotic surgeons. While it has been demonstrated that curricula improve technical performance, less is known about resident factors that predispose poor baseline performance and whether structured training can reduce these disparities. Predictors of early underperformance were hypothesized to be attenuated after completion of a multimodal robotic curriculum.

**Methods:**

PGY-3 general surgery residents (2019–2023) completed a standardized 2-week curriculum. Training included periodic assessment tests, virtual reality (VR) simulation training, and procedure-based simulation with bio-tissue drills. Objective performance across the curriculum was evaluated, and resident-specific predictors of performance were examined. Performance was graded by an expert surgeon using OSATS (range 6–30). Perceived mental workload and stress were measured using the NASA Task Load Index, Borg Exertion Scale, and Edwards Arousal Rating.

**Results:**

Thirty-nine residents completed the curriculum. Residents were stratified by pre-test mOSATS; high (top 25%), mid (50%), and low (bottom 25%). Pre-test strata remained associated with VR post-test strata (50% of high performers and 55% of low performers remained in pre-test strata; *p* = 0.041). However, pre-test and post-test strata were not associated with final test performance after bio-tissue drills. Higher pre-curriculum comfort predicted high pre-test performance (*p* = 0.003). Low pre-test performance was associated with male sex (*p* = 0.010), less robotic experience (*p* = 0.025), and lower pre-curriculum comfort (*p* = 0.031). Anticipated career use of robotics predicted better initial bio-tissue drill performance (running hepaticojejunostomy *p* = 0.042; interrupted hepaticojejunostomy *p* = 0.024). Higher baseline stress correlated with worse final pancreatojejunostomy performance (Spearman *ρ* = −0.46; *p* = 0.004).

**Conclusions:**

Resident characteristics influence early robotic simulation performance, but a structured curriculum mitigates baseline disparities such that initial performance and its predictors no longer influence final outcomes. These findings highlight the value of a robotic curriculum’s ability to foster competency and optimize resident training.

During the last two decades, robotic surgery has become the dominant minimally invasive operative platform across multiple surgical specialties with the primary displacement of laparoscopic procedures [1, 2]. With the continued advancement of minimally invasive surgery, proficiency in robotic surgery has become necessary for aspiring young surgeons as residents are now exposed to this platform earlier in training. New expectations and pressure for graduating residents to achieve robotic competency have emerged despite wide variability in intraoperative console exposure and autonomy [3–5].

Surgical education has historically addressed similar gaps with laparoscopy and endoscopy through standardized, competency-based training models, such as fundamentals of laparoscopic surgery (FLS) and fundamentals of endoscopic surgery (FES). Early completion of these training models has been associated with measurable improvements in autonomy, operative, and procedural performance by emphasizing deliberate practice, objective assessment, and proficiency prior to clinical application [6–8]. Fundamentals of Robotic Surgery (FRS) model is a validated national robotic skills curriculum, but it has not been scaled broadly and is not mandated across residency programs [9]. Currently, the Accreditation Council for Graduate Medical Education (ACGME) does not have a recognized standardized robotic curriculum requirement in surgery residency training despite the growing demands to efficiently produce excellent and confident robotic surgeons upon residency graduation. Furthermore, in the modern era, utilizing supervised patient care experiences as models for practice is no longer acceptable when practice through simulation has repeatedly demonstrated efficacy in the rate at which skill is acquired [10–12].

Previous studies demonstrate that curricula involving VR simulation and inanimate training can improve overall performance; however, it is unclear which resident factors differentiate early performance and whether these baseline differences persist after completing a formal training curriculum [13–16]. Understanding predictors of performance has direct implications for program design, including how early curricula should be implemented, which residents may benefit from targeted support, and whether training can mitigate disparities created by variable operative and clinical exposure. A previously described standardized two-week robotic curriculum was implemented for general surgery residents [17]. Identifiable resident factors were hypothesized to be associated with early performance, but we believed that completion of a structured curriculum would attenuate these differences, resulting in comparable performance at the end of training.

## Materials and methods

### Study design and participant selection

This IRB-approved study was a retrospective analysis of prospectively collected data from the University of Chicago general surgery residents from 2019 to 2023. Training and assessment were conducted at the Grainger Center for Simulation and Innovation at Endeavor Health, Evanston Hospital. Participating general surgery residents provided informed consent prior to participation. Data were collected in accordance with IRB regulations, stored on secure, access-restricted institutional systems, and de-identified prior to analysis. This observational study was reported in accordance with the Strengthening the Reporting of Observational Studies in Epidemiology (STROBE) guidelines.

### Robotic surgery curriculum

General surgery residents completed a mandatory standardized two-week curriculum on the daVinci Surgical System platform (Intuitive Surgical Inc, Sunnyvale, CA, USA) consisting of three phases of training: (1) virtual reality (VR) simulation, (2) technical drills, including suturing and knot tying exercises, and (3) procedure-based simulation drills using inanimate bio-tissue and replica models [17, 18]. During the curriculum, an individualized mentor session with an experienced robotic surgeon was held, and three identical proficiency assessment tests were completed.

Prior to starting the first phase of the robotic surgery curriculum, each participant completed a pre-test to establish baseline assessment of skill. The pre-test consisted of four VR exercises and three technical skill exercises on the daVinci Xi simulator. The first phase of the curriculum required completion of 43 VR simulation exercises on the Si/Xi daVinci backpack. Demonstration of proficiency was required prior to participant advancement and was defined as achieving > 90% or completing 10 attempts on each VR simulation task. Participants then completed a post-VR simulation assessment test that consisted of the same four VR exercises and three technical skill exercises as the pre-VR test.

The second phase of the curriculum consisted of fundamental robotic suturing and knot tying drills on the daVinci Xi platform. Following completion of the suturing and knot tying drills, a one-on-one mentor session was held with an experienced robotic surgeon. Once adequate proficiency was ensured, participants moved on to the third and final phase of the curriculum which consisted of procedure-based simulation drills: (1) inguinal and ventral hernia repair and (2) bio-tissue drills designed to replicate the anastomoses of a robotic pancreaticoduodenectomy. The bio-tissue drills were completed in order of classically perceived technical difficulty and consisted of (1) running hepaticojejunostomy (RHJ), (2) gastrojejunostomy (GJ), (3) interrupted hepaticojejunostomy (IHJ), (4) and pancreatojejunostomy (PJ). Each simulation drill was performed four times sequentially before moving to the next drill. The final periodic assessment test was performed after completion of all VR simulation tasks, technical drills, 1:1 mentorship, and procedure-based simulation modules. This assessment was identical in structure to the pre-VR and post-VR periodic assessments, consisting of four VR exercises and three dry lab technical skills exercises.

### Participant performance and video-based assessment

The procedure-based simulation drills and periodic assessment tests (i.e., pre-VR, post-VR, and final test) underwent video-based assessment by a single experienced surgeon. Each participant’s assessment tests and drills were graded retrospectively. The video grader was blinded and only received de-identified videos. A modified version of the Objective Structured Assessment of Technical Skills (mOSATS) tailored to robotic surgery was utilized [19]. While GEARS is a modern externally validated robotic surgery assessment tool, mOSATS was utilized for assessment to maintain consistency with the previously validated curriculum.

Participants were assessed and graded based on gentleness and respect for tissue (1–5), efficiency of time and motion (1–5), adequacy of instrument handling (1–5), sustained organized flow of operation (1–5), appropriate tissue exposure (1–5), and overall summary of technical skill (1–5), for a total composite OSATS score of 6–30. The three periodic assessment tests consisted of three scores: (1) total VR simulation score, (2) total time spent on the dry lab technical skills component, and (3) average mOSATS score of the technical skills component. Each video of the four recorded sessions for the ventral hernia, inguinal hernia, RHJ, GJ, IHJ, and PJ component of the curriculum was similarly graded using mOSATS.

### Participant self-assessment and surveys

Prior to starting the curriculum, residents completed an intake survey which included general demographic information, previous surgical experience, and future career plans. Several self-assessment surveys were completed by participants at key transition phases of the curriculum. These interval questionnaires consisted of The NASA Task Load Index (NASA-TLX), Borg Exertion Scale, and The Edwards Arousal Rating [20–22]. Task surveys were used to reliably assess participant self-assessment of performance and mental workload. The NASA-TLX consists of six domains including mental demand, physical demand, temporal demand, perceived effort, perceived performance, and frustration while performing the task. Each domain was rated on a 20-point scale (0–20) with 0 indicating very low and 20 indicating very high for a total of 120 points. Higher scores were indicative of perceived worse performance and greater mental workload. The Edwards Arousal Rating used a 7-point Likert scale which assessed nervousness, fear, and anxiety. The Borg Exertion Scale was used to measure perceived physical exertion and scored on a scale of 0–10 with 0 indicating no exertion at all and 10 indicating maximal exertion. Residents completed a long-term, post-curriculum follow-up to assess performance and confidence after the curriculum.

### Statistical analysis

All statistical analyses were performed using SAS 9.4 (SAS Institute, Cary, NC, USA). Participants were stratified into performance tiers based on periodic mOSATS assessments: high (top 25%), mid (middle 50%), and low (bottom 25%). Normally distributed continuous variables are reported as mean with standard deviation (SD), and categorical variables are reported as frequency (percentage). The primary analysis was directed toward participant initial assessment test performance and the association with final assessment test performance. Associations involving demographic characteristics, subjective workload measures, and individual performance on procedure-based drills were exploratory and not adjusted for multiple comparisons. Differences in continuous outcomes across performance tiers were evaluated using one-way analysis of variance (ANOVA), with post hoc pairwise comparisons performed when appropriate. Associations between objective performance metrics and subjective workload measures were assessed using Spearman’s rank correlation coefficient (*ρ*). Changes in participant performance across curriculum phases were evaluated using paired *t*-tests. Statistical significance was defined as *p* < 0.05.

## Results

### Cohort baseline characteristics and survey participation

Thirty-nine general surgery residents enrolled in the curriculum from 2019 to 2023. All residents successfully completed the curriculum. Task-specific and curriculum intake surveys were completed entirely by 38/39 (97.4%) of residents. The average reported age of participating residents was 31 ± 2 years old. The curriculum was completed by 35 (89.7%) residents during PGY-3, and the remaining 4 (10.3%) residents completed the curriculum during PGY-4 or dedicated research years. All 39 (100%) residents believed that training in robotic surgery was an essential part of general surgery training. 30 residents (76.9%) agreed that a formal robotic curriculum should be included in general surgery training. In addition, 30 residents (76.9%) anticipated using robotic surgery in future practice. Of those who advocated for a formal curriculum in robotic surgery, 22 (73.3%) expected to use robotic surgery in future practice. 29 (74.4%) participants previously practiced using the daVinci Xi backpack simulator and 33 (84.6%) residents indicated that they had previous console experience in the operating room. The average number of robotic surgery cases performed prior to starting the curriculum was 10.4 ± 11.6. Previous experience assisting laparoscopically for robotic surgery cases was reported by 37 (94.9%) residents with 12.6 ± 9.0 mean number of cases by each resident.

### Curriculum performance outcomes

**Fig. 1:**
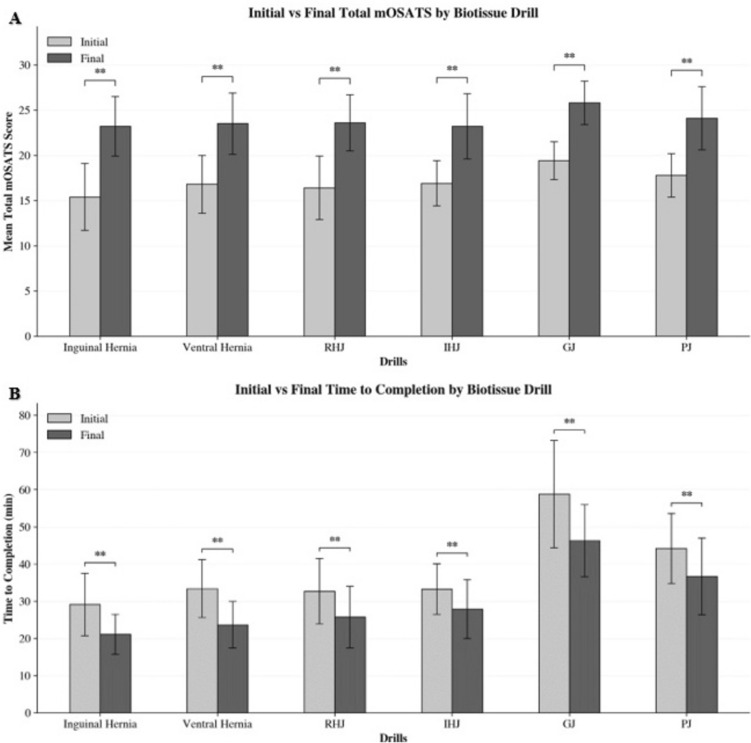
**A** Mean total mOSATS and **B** time to completion for procedure-specific drills. Error bars represent standard deviation. Brackets indicate paired comparisons between initial and final attempts; *p-value < 0.05; **p-value < 0.001. *mOSATS* modified Objective Structured Assessment of Technical Skill, *RHJ* running hepaticojejunostomy, *IHJ* interrupted hepaticojejunostomy, *GJ* gastrojejunostomy, *PJ* pancreatojejunostomy

Periodic assessment tests were administered at three critical time points during the curriculum to evaluate performance changes across VR training and procedure-based simulation drills. Periodic assessment test performance improved across the curriculum. Pre-assessment (i.e., before VR simulation and procedure drills) showed a mean composite VR score of 161 ± 41 (Maximum 400), mean total mOSATS of technical skill exercises 15.8 ± 3.3, and mean total time to completion of technical skill exercises 1144 ± 387 s. Following completion of the VR simulation phase of the curriculum, the mean composite VR score increased to 228 ± 28 (*p* < 0.001 vs. pre-assessment), mean total mOSATS of technical skill exercises increased to 20.9 ± 2.2 (*p* < 0.001 vs. pre-assessment), and time to completion of technical exercises decreased to 796 ± 207 s (*p* < 0.001). Following successful completion of all curriculum drills, the final test demonstrated a mean composite VR score that further increased to 236 ± 36 (*p* < 0.001 vs. pre-assessment; *p* = 0.030 vs. post-VR assessment), mean mOSATS increased to 25.7 ± 2.2 (*p* < 0.001 vs. pre-assessment; *p* < 0.001 vs. post-VR assessment), and time to completion decreased to 494 ± 129 s (*p* < 0.001 vs. pre-assessment; *p* < 0.001 vs. post-VR assessment). Regardless of procedure-based simulation drill performed, residents consistently demonstrated significant improvement in total mOSATS score (Fig. 1A) and reduced time to completion (Fig. 1B) of assigned task when comparing the initial to final attempt.

### Predictors of curriculum performance

**Fig. 2:**
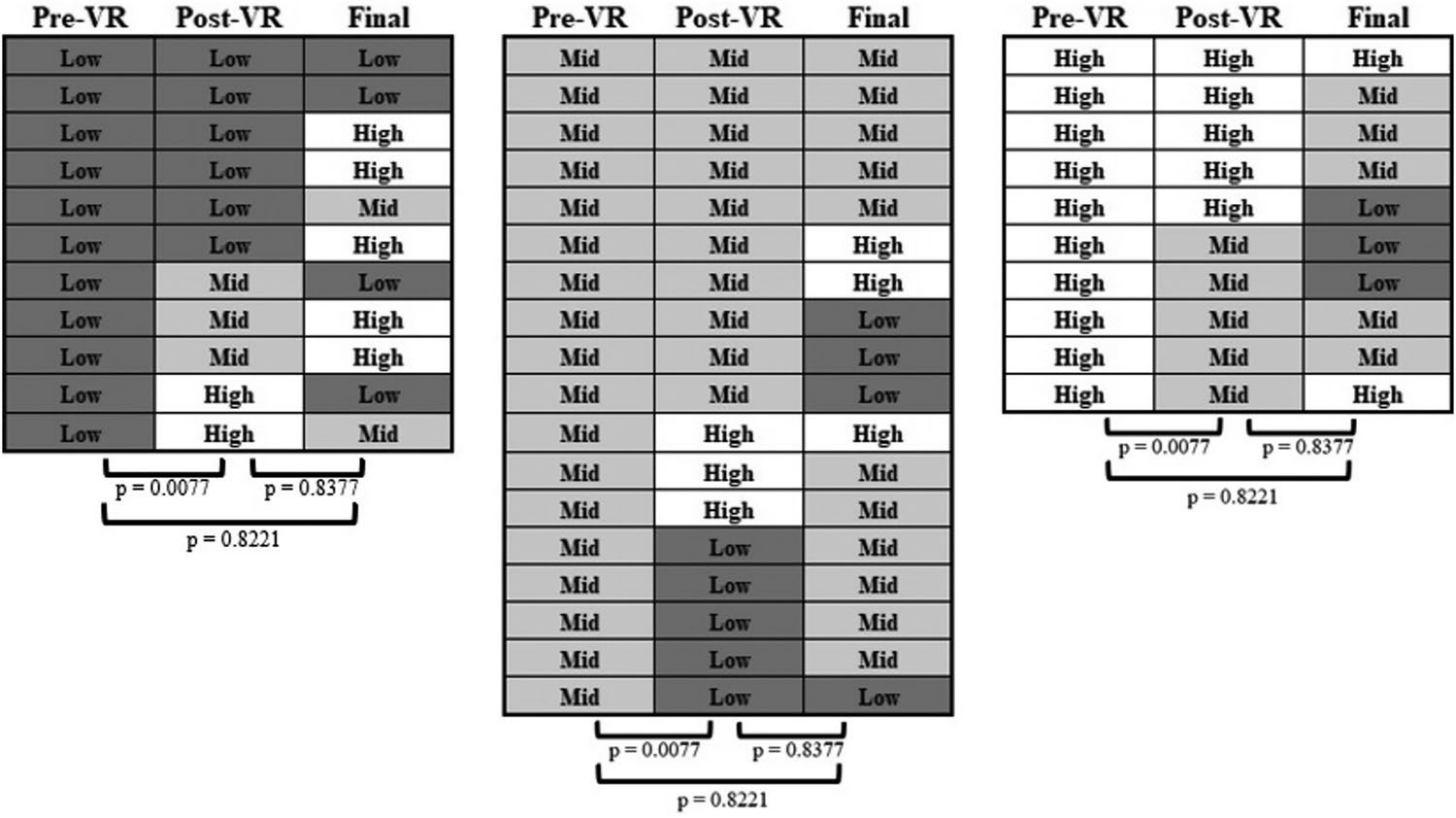
Individual resident periodic assessment test performance across the curriculum, stratified by baseline pre-VR performance tier (i.e., pre-curriculum test). Residents are grouped as low, mid, or high performers at pre-VR and then followed longitudinally through post-VR and final assessment tests

Residents were divided into three groups based on pre-test OSATS performance: high (top 25%), mid (middle 50%), and low (bottom 25%) performers (Fig. 2). 50% of high performers on the pre-VR test remained high performers on the post-VR test. Similarly, 55% of low performers on the pre-test were low performers on the post-VR test (*p* = 0.008). However, performance on the final test after completion of the curriculum was not associated with performance on the pre-VR test (*p* = 0.822) and post-VR test (*p* = 0.8377). Of the high performers at the final assessment test, 50% were initially low performers on the pre-VR test at the start of the curriculum, while only 20% were initially high performers.

Previous operating room robotic console experience was associated with significantly higher pre-VR assessment test performance, with demonstration of higher baseline scores across all tested metrics as the number of prior robotic cases performed increased (Table 1). Residents with fewer prior robotic cases demonstrated greater improvement (Δ) across metrics, whereas those with ≥ 10 prior cases showed smaller gains. However, after curriculum completion, prior console experience was no longer predictive of performance on the final periodic assessment test. Similarly, once participants were stratified by pre- and final test performance, prior robotic case experience was not predictive of high performance on the pre-test (*p* = 0.220) or the final test (*p* = 0.094).

**Table 1:** Assessment test performance by number of previous robotic surgery cases performed

		Number of previous robotic surgery cases performed			*p*-value
	All (*N* = 38)	0–2 Cases (*N* = 11)	3–9 Cases (*N* = 15)	≥ 10 Cases (*N* = 12)	–
Composite VR score (mean ± SD)					
Pre-VR test	163 ± 40	144 ± 30	150 ± 38	195 ± 32	0.0019
Final test	228 ± 29	228 ± 29	222 ± 34	235 ± 22	0.6488
Δ Total score (final minus pre)	65 ± 37	84 ± 36	72 ± 34	40 ± 32	0.0113
Mean mOSATS of technical exercises (Mean ± SD)					
Pre-VR test	15.9 ± 3.3	13.7 ± 2.0	15.7 ± 3.6	18.1 ± 2.3	0.0040
Final test	21.0 ± 2.2	20.0 ± 2.4	21.6 ± 1.7	21.2 ± 2.2	0.2743
Δ OSATS (final minus pre)	5.1 ± 2.9	6.4 ± 2.7	5.9 ± 2.9	3.1 ± 1.7	0.0038
Total time of technical exercises (seconds)					
Pre-VR test	1149 ± 391	1349 ± 386	1242 ± 365	849 ± 243	0.0026
Final test	794 ± 209	854 ± 240	815 ± 196	711 ± 183	0.2930
Δ Time (final minus pre)	− 355 ± 336	− 496 ± 416	− 427 ± 279	− 137 ± 211	0.0045

Residents who anticipated using robotics in future practice had higher mOSATS scores on the initial RHJ and IHJ bio-tissue drills (RHJ, *p* = 0.042; IHJ, *p* = 0.024) and on the final IHJ drill (*p* = 0.043), but no difference was observed for periodic assessment tests or the remaining drills (inguinal hernia, ventral hernia, final RHJ, GJ, and PJ; all other comparisons* p* > 0.05). Residents who were high performers on the pre-test were younger (Mean age 29.6 ± 1.5 vs. 31.4 ± 1.8; *p* = 0.007), left-handed/ambidextrous (*N* = 4; *p* = 0.025), and reported higher comfort level with the robot prior to the curriculum (*p* = 0.003). Residents who were low performers were more likely to be male (*p* = 0.010), had less experience (*p* = 0.025), and reported less comfort with the robot prior to the curriculum (*p* = 0.031). However, handedness, age, gender, and experience were no longer predictive of performance at the end of the curriculum on the final assessment test (*p* > 0.05).

### Participant self-assessment of performance

At the final periodic assessment test, residents reported improved comfort as demonstrated by reduced Edwards Arousal Rating, NASA-TLX, and Borg Exertion Scale scores compared with the pre-test (Edwards Arousal Rating: 8.1 ± 3.0 vs. 9.9 ± 3.5, *p* = 0.010; NASA-TLX: 49.6 ± 12.8 vs. 70.8 ± 17.9, *p* < 0.001; Borg Exertion Scale: 1.3 ± 0.9 vs. 2.6 ± 1.0, *p* < 0.001). On the NASA-TLX, residents with high console experience were more likely to rate performance during the curriculum more favorably (10.3 vs. 14.8, *p* = 0.017), feel less frustrated (11.4 vs. 14.5, *p* = 0.007), and feel as if they exerted less effort (9.8 vs. 14.3,* p* = 0.019) compared to those with low experience.

When stratified by pre-test performance, high pre-test performers demonstrated lower NASA-TLX scores compared to low and middle performers (57.7 ± 12.1, *p* = 0.001), but this difference between groups was not seen after completion of the curriculum during the final assessment (*p* = 0.063). Furthermore, at the completion of the curriculum, no difference was noted between high, mid, and low final test performers when assessing for mental load, stress, and exertion level during the final test (Edwards Arousal Rating: *p* = 0.229; NASA-TLX: *p* = 0.630; Borg Exertion Scale: *p* = 0.844).

Across the pre-test, higher perceived arousal and workload were associated with worse objective performance (Table 2). On the pre-test, Edwards Arousal ratings correlated with lower composite VR scores, longer total task time, and lower mean mOSATS, while NASA-TLX showed a similar inverse relationship with mean mOSATS. By the final periodic assessment, these associations were no longer observed, with no significant correlation between survey measures and composite VR score, total time, or mean mOSATS. When assessing bio-tissue drill performance, higher perceived workload was linked to lower first-attempt performance for select tasks (RHJ and PJ), but these relationships did not persist on final-attempt mOSATS scores.

**Table 2:** Correlation of self-reported resident mental task load assessment and curriculum performance

	Task survey		
	Edwards arousal rating sum	NASA-TLX sum	Borg exertion scale
Pre-test			
Composite VR score	−0.32**	−0.31*	−0.14
Total time of technical exercises	0.39**	0.18	0.13
Mean mOSATS of technical exercises	−0.38**	−0.46**	−0.18
Final test			
Composite VR score	−0.03	−0.06	−0.05
Total time of technical exercises	−0.21	−0.21	−0.05
Mean mOSATS of technical exercises	0.27	0.16	−0.05
Running hepaticojejunostomy			
First attempt mOSATS	−0.42**	−0.22	−0.18
Final attempt mOSATS	−0.26	−0.13	−0.16
Interrupted hepaticojejunostomy			
First attempt mOSATS	−0.22	0.01	0.04
Final attempt mOSATS	−0.13	0.16	0.16
Gastrojejunostomy			
First attempt mOSATS	0.21	−0.01	−0.03
Final attempt mOSATS	0.02	−0.04	0.08
Pancreaticojejunostomy			
First attempt mOSATS	−0.19	−0.35**	−0.37**
Final attempt mOSATS	−0.19	−0.17	−0.05

Spearman’s rank correlation coefficients (*ρ*) between objective performance metrics (pre-test, final test, and bio-tissue drill performance) and task survey scores (Edwards Arousal rating, NASA-TLX, and Borg Exertion Scale)

^*^ρ ≥ 0.30 but *p* > 0.05 (moderate correlation, not statistically significant)

^**^p < 0.05

### Follow-up outcomes and resident perceived skill change

A post-curriculum follow-up survey was completed by 19/39 curriculum participants. Residents’ self-reported robotic skill changes over time since completion of the robotic curriculum varied by subsequent robotic case volume (Table 3). 9/19 (47.4%) reported diminished skill, 2/19 (10.5%) reported no change, 6/19 (31.6%) reported slight improvement (101–200%), and 2/19 (10.5%) reported significant improvement (> 200%). Greater post-curriculum case volume was moderately correlated with perceived improvement (*r* = 0.52, *p* = 0.020), and lack of robotic volume was the most common explanation for perceived skill degradation. Overall robotic confidence declined over time, with 13 residents reporting “slightly/very confident” at follow-up compared with 18 residents immediately after curriculum completion.

**Table 3:** Resident self-reported degree of robotic skill change since curriculum completion, stratified by number of robotic cases performed post-curriculum

Perceived skill since completion of curriculum (% of skill change)	Number of residents *N* = 19 (% of total residents)	Residents by number of cases performed after curriculum (% within each case volume group)				
		0 Cases (*N* = 3)	1-5 Cases (*N* = 2)	6-20 Cases (*N* = 6)	21-50 Cases (*N* = 7)	> 51 Cases (*N* = 1)
Diminished (< 100%)	9 (47.4%)	1 (33.3%)	2 (100%)	4 (66.7%)	2 (28.6%)	0 (0.0%)
No change (100%)	2 (10.5%)	2 (66.7%)	0 (0.0%)	0 (0.0%)	0 (0.0%)	0 (0.0%)
Slightly improved (101–200%)	6 (31.6%)	0 (0.0%)	0 (0.0%)	2 (33.3%)	4 (57.1%)	0 (0.0%)
Significantly improved (> 200%)	2 (10.5%)	0 (0.0%)	0 (0.0%)	0 (0.0%)	1 (14.3%)	1 (100%)

Percent skill change was reported relative to skill at curriculum completion (100%)

## Discussion

Our results suggest that a structured, practice-based robotic simulation curriculum can improve technical performance and reduce baseline disparities among residents, resulting in comparable outcomes at the end of the curriculum. Early predictors of poor baseline performance included male sex, lower prior experience, lower console comfort, and higher perceived stress, whereas left-handedness and prior console experience were associated with higher baseline performance. However, pre-test performance was no longer predictive of final assessment performance after curriculum completion, and resident-level predictors that influenced baseline performance were no longer associated with end-of-curriculum outcomes. Subjective workload measures aligned with objective performance at baseline for select tasks, but these relationships diminished by curriculum completion, consistent with reduced cognitive burden as skills strengthened.

The observed improvement from low baseline performance to high final performance underscores the value of repeated practice with objective assessment. Half of the final high performers started in the low-performance tier at pre-VR, and only one-fifth were high performers at baseline. This supports a training approach that prioritizes early identification of learners who need more repetition and coaching, rather than relying on baseline performance to forecast end-of-training readiness. Overall, completion of the curriculum appeared to normalize performance even among residents with higher baseline stress and performance anxiety, supporting the curriculum’s ability to equalize competency and optimize resident performance.

These findings are consistent with the broader surgical education literature demonstrating that simulation-based, proficiency-oriented training can accelerate acquisition of minimally invasive technical skills and improve objective performance metrics [14, 16, 17]. Standardized competency-based models in laparoscopy and endoscopy, such as FLS and FES, have reinforced the value of deliberate practice and objective assessment prior to clinical application. As previously described, robotic curricula that incorporate VR training, technical drills, and procedure-based simulation similarly report improvements in technical skill and efficiency during simulation-based testing. What has remained less clear is whether structured curricula primarily benefit residents who enter with greater exposure or whether they can reduce baseline disparities created by variable operative opportunity. Our data suggest that while informal console exposure confers an early advantage, a standardized curriculum can attenuate these baseline differences by the end of a structured curriculum, supporting simulation as an equitable mechanism for skill acquisition when real-world exposure is inconsistent.

Some published experiences imply that early high performers remain high performers throughout training or that prior operative exposure reliably predicts downstream robotic competency [23]. In contrast, our results suggest that baseline predictors, including pre-test performance, do not necessarily persist following completion of a structured curriculum. This difference may reflect the intensity and standardization of the training intervention, repeated assessments across defined time points, and the emphasis on platform-specific fundamentals that may be rapidly improved through deliberate practice. Early associations between subjective workload and objective performance diminished by curriculum completion, suggesting that stress and cognitive load may be most influential during the novice phase and become less relevant as residents gain skill and efficiency. These patterns support a practical design principle for robotic education: curricula should identify baseline variability and reliably bring residents to a common competency threshold through repeated, objectively assessed practice.

Residency training, given the length of time and heterogeneity among rotations, poses a larger challenge for robotic training than fellowship. While a formal robotic curriculum provides necessary confidence and skills, the lasting impact may be diminished by subsequent limited robotic exposure and autonomy. A national survey of general surgery programs suggests that formal robotic curricula are increasingly common, yet meaningful resident console autonomy remains limited [24]. In a 2022 survey of residency programs, 70% of respondent programs reported having a structured robotic surgery curriculum, but 45% of programs reported that operating on the robotic surgery console began at the PGY-3 level, with most programs reporting that senior level residents spent less than half of the operative time at the console. Notably, the majority of curricula had been implemented for less than 3 years at the time of this study, so limited resident autonomy likely reflects multiple factors related to novelty and inexperience [25]. Nonetheless, restricted console time is detrimental to skill acquisition for surgeons in training and may not be justified based on studies demonstrating similar total operative time and patient outcomes when resident autonomy is encouraged [26–28]. This reinforces the need for standardized training pathways that build baseline proficiency and facilitate graduated autonomy in the operating room. Additionally, reducing resident stress and anxiety in simulation by acquisition of skills can have positive effects on intraoperative performance and is a new avenue of investigation.

This study had several limitations. An inherent limitation is that this was a single-institution experience with a small cohort of participants, and outcomes that may reflect local teaching practices at the University of Chicago, simulator environment, and curriculum structure. Furthermore, this curriculum requires a dedicated simulation center, access to experienced mentors, two weeks of protected resident education time, and resources that may not be available to all programs. This limits generalizability and reproducibility on the national scale. However, future integration of similar curricula will require customized approaches based on resources available.

A second limitation is that the primary outcomes were simulation-based performance metrics; however, there is emerging evidence to suggest that intraoperative video grading with mOSATS may transition to predictable patient outcomes, and that performance in simulation translates to post-operative outcomes [15, 29–31]. An additional limitation is the absence of GEARS as an assessment tool and the lack of multi-evaluator assessment, which introduces potential bias. Future studies may benefit from utilizing both mOSATS and GEARS and from introducing more than one grader for resident evaluation to ensure consistency and generalizability.

Moreover, our findings also suggest that VR simulation performance does not reliably translate to procedure-based proficiency. The largest standardization in performance occurred after residents completed technical drills and bio-tissue modules rather than after completing the VR portion of the curriculum. This suggests that VR simulation may be more important for establishing robotic surgery console familiarity and fundamental technique rather than being predictive of long-term performance and may not translate to operative room performance. These data suggest a benefit to introducing procedure-based simulation earlier during surgical training, and using VR as an adjunct for foundational console mechanics rather than as the primary gatekeeping determinant of proficiency.

Finally, long-term conclusions regarding acquired skill durability were constrained by variable post-curriculum operative exposure, incomplete follow-up, and primarily relying on self-reported measures of skill retention. Long-term skill retention findings were derived from self-reported surveys, completed by 19/39 residents, and not practically based on objective metrics of performance; therefore, long-term findings should be interpreted cautiously. Despite these limitations, the consistency of performance improvement and the attenuation of baseline predictors after simulation training support the educational value of a structured robotic curriculum.

In conclusion, a structured two-week robotic curriculum incorporating VR training and procedure-based simulation improved objective performance and reduced baseline disparities among PGY-3 residents, such that early predictors of performance were less relevant by the final assessment. A key application of this work is to pair early simulation-based robotic training with deliberate post-curriculum consolidation, including intentional integration as console surgeon, scheduling of high-volume robotic rotations soon after curriculum completion, and structured skills maintenance outside the operating room, to preserve proficiency and support graduated autonomy.

More broadly, these data support continued movement toward standardized competency-based training in robotic-assisted surgery. Although implementation of a standardized curriculum remains logistically challenging, successful adoption will require flexibility across programs with different resources and training structures. As a future direction, an ACGME standardized fundamentals of surgery rotation introduced early in residency and incorporating open, laparoscopic, and robotic training may help establish a national baseline for technical development, improve resident preparedness, and guide progression toward independent practice.
