# Correction: Balancing the scales: predictors of performance and the long-term impact of a robotic surgery curriculum

**DOI:** 10.1007/s00464-026-13184-8

**Published:** 2026-08-03

**Authors:** Colin M. Johnson, Sarah B. Hays, Jason L. Schwarz, Kristine Kuchta, Aram Rojas, Syed A. Mehdi, Sangrag Ganguli, Alessia Vallorani, Miral S. Grandhi, Melissa E. Hogg

**Affiliations:** 1 Department of Surgery, Rutgers New Jersey Medical School, Newark, NJ USA; 2 Department of Surgery, University of Chicago, Chicago, IL USA; 3 Department of Surgery, Endeavor Health – Evanston Hospital, 2650 Ridge Ave, Chicago, IL 60201 USA; 4 Division of Pancreatic and Transplant Surgery, Pancreas Translational and Clinical Research Center, IRCCS San Raffaele Scientific Institute, Milan, Italy

**Correction to: Surgical Endoscopy** **https://doi.org/10.1007/s00464-026-13035-6**

The original online version of this article was revised to correct the following passage in the Disclosures section:

Dr. Melissa E. Hogg receives funding from Intuitive Surgical for observations, engaging in HPB courses, and proctoring, as well as Olympus where she is a consultant, but there is no direct funds for > 2 years.

The article was also updated to denote the following affiliation for coauthor Alessia Vallorani:

Division of Pancreatic and Transplant Surgery, Pancreas Translational and Clinical Research Center, IRCCS San Raffaele Scientific Institute, Milan, Italy.

The original article has been corrected.

